# A Cluster Analysis of EPOCH Questionnaire Data from University Students in Sichuan, China: Exploring Group Differences in Psychological Well-Being and Demographic Factors

**DOI:** 10.3390/healthcare13192476

**Published:** 2025-09-29

**Authors:** Juan Wan, Lijuan Ren, Yufei Tan, Yin How Wong, Ching Sin Siau, Lei Hum Wee

**Affiliations:** 1 School of Software Engineering, Chengdu University of Information Technology, Chengdu 610225, China; 2 Centre for Community Health Studies, Faculty of Health Sciences, University Kebangsaan Malaysia, Kuala Lumpur 50300, Malaysia; 3 School of Medicine, Faculty of Health and Medical Sciences, Taylor’s University, Subang Jaya 47500, Malaysia; 4 Digital Health and Medical Advancement Impact Lab, Taylor’s University, Subang Jaya 47500, Malaysia

**Keywords:** EPOCH, K-means clustering, psychological well-being, university students, China

## Abstract

(1) Background: University students face increasing mental health challenges, with sociodemographic disparities shaping well-being outcomes and highlighting the need for machine learning approaches to identify distinct psychological profiles. (2) Methods: This cross-sectional study surveyed 4911 Chinese university students (aged 18–25) using the EPOCH Questionnaire, which measures Engagement, Perseverance, Optimism, Connectedness, and Happiness. Data were collected via WenjuanXing (WJX), with recruitment promoted through official channels. Well-being profiles were identified through exploratory K-means clustering, with internal validity and the optimal cluster number assessed using the silhouette coefficient. (3) Results: Cluster analysis identified two distinct groups: Cluster 0 (41.09%) with higher well-being scores and Cluster 1 (58.91%) with lower scores. Differences across all five EPOCH dimensions exceeded 1.0, most notably in Optimism (Δ = 1.31) and Happiness (Δ = 1.37). A subgroup of concern within Cluster 1 (*n* = 92), primarily male sophomores from rural, low-income, multi-child families receiving financial aid, showed particularly low scores in Connectedness (Δ = −0.57) and Happiness (Δ = −0.43). In contrast, a high well-being subgroup in Cluster 0 (n = 108), mainly urban female freshmen from high-income, only-child families, exhibited elevated scores, especially in Connectedness (Δ = 0.69) and Happiness (Δ = 0.65). (4) Conclusions: This exploratory clustering study identified distinct well-being profiles among Chinese university students, with demographic and socioeconomic vulnerabilities associated with diminished psychological well-being, particularly in Connectedness, Happiness, and Optimism. These findings highlight the need for targeted interventions that integrate psychosocial support with financial assistance to reduce inequalities and promote flourishing.

## 1. Introduction

Mental health issues among university students have attracted increasing attention because of their high prevalence and negative impact on academic performance, social relationships, and overall quality of life [1]. Universities worldwide have noted reductions in student well-being [2,3]. Studies also indicate rising rates of depression, anxiety, and stress among students [4]. Given this continued deterioration in mental health, university student well-being remains a key concern [3].

Well-being is a multidimensional construct that includes mental health, life satisfaction, purpose, and stress management [5]. It encompasses both hedonia, often reflected in subjective well-being (SWB), and eudaimonia, captured by psychological well-being (PWB). Although distinct, these constructs overlap, and evidence from SWB research informs the study of PWB. Studies show that sociodemographic factors influence well-being differently across contexts, with urban populations benefiting more from social participation and rural populations relying on trust and reciprocity [6,7,8]. Recent evidence also indicates that the spatial effect of social ties is stronger in rural areas than in urban areas [9]. Overall, gender, family structure, income, and urban–rural differences are essential for understanding student mental health and well-being [10,11,12].

The engagement, perseverance, optimism, connectedness, and happiness (EPOCH) model was developed by Kern et al. in 2016 [13] to capture positive psychological functioning in youth. It highlights the role of personal strengths that foster both subjective and psychological well-being, and it is conceptually grounded in well-being theory. Empirical research has consistently supported the factor structure of the EPOCH model across diverse cultural contexts. In particular, validations of the Chinese version, based on large samples of university students, confirmed its reliability and applicability in 2019 [14].

Empirical evidence indicates that the EPOCH domains are associated with both psychological outcomes and sociodemographic factors. The overall scale and its subscales have been linked to depression, anxiety, behavior problems, delinquency, school connectedness, and social skills in adolescents [15]. Validations of the EPOCH domains demonstrate scalar invariance across gender and age, supporting meaningful comparisons of latent means between demographic groups [13,16]. Evidence from Chinese student samples in 2019 further confirms the measure’s applicability across gender groups [17], while studies in Austria also reported invariance across gender and age [16]. More recent cross-cultural validations provide additional support. The Sinhala adaptation proved reliable in assessing the five dimensions of well-being among adolescents in Sri Lanka [18]. Research in India also reported gender-related differences, with higher academic buoyancy among males and greater connectedness among females [19].

Most earlier studies used traditional statistical techniques to analyze EPOCH data, focusing on overall trends at the group level [17,20,21]. However, these methods often fail to detect latent psychological patterns or identify meaningful individual differences. Recent advancements in data mining, particularly the adoption of clustering algorithms, have allowed for more nuanced, data-driven investigations into mental health heterogeneity [22,23]. The use of machine learning has become increasingly prominent in bioinformatics and interdisciplinary research [24,25,26]. In the standard K-means algorithm, the number of clusters must be specified by the user, with initial centers selected arbitrarily from the dataset [27]. Its simplicity, scalability, and effectiveness in handling multidimensional psychological data make it particularly valuable [28]. Recent applications of K-means have identified subgroups based on psychological profiles. These findings provide evidence for the development of tailored mental health strategies. For example, Liu applied an improved K-means algorithm and revealed gender-based differences in mental well-being [29]. Research on college students has also employed clustering to investigate mental health education [30], and Septiadi [31] used K-means to classify sleep patterns and health metrics. These applications illustrate the value of clustering methods in detecting heterogeneity in psychological outcomes. At the same time, sociodemographic factors such as gender, socioeconomic background, and family structure are increasingly recognized as interacting with psychological characteristics in complex ways [32,33,34], underscoring the need for integrative analysis.

Although previous studies have advanced the understanding of student well-being, the ways in which psychological and sociodemographic factors combine to form distinct well-being profiles remain underexplored [7,8,9,10,11,12]. Building on prior validations, the present study does not re-examine the psychometric properties of the EPOCH scale but instead applies it as an established framework to explore latent clusters of well-being among Chinese university students. Using K-means clustering, we investigate how engagement, perseverance, optimism, connectedness, and happiness cluster within this population and how these clusters vary across gender, family structure, socioeconomic background, and urban–rural context. By adopting a person-centered and application-focused approach, this study extends the utility of the EPOCH framework and provides evidence to inform tailored mental health strategies in higher education. Consistent with prior research highlighting heterogeneity in student well-being, we hypothesize that distinct latent clusters of psychological well-being will emerge among Chinese university students, differentiated by both EPOCH dimensions and sociodemographic characteristics.

## 2. Materials and Methods

### 2.1. Data Collection

Convenience sampling was employed for its practicality and efficiency in accessing diverse participants [35], as demonstrated in recent studies on workplace violence among nurses [36], resilience and lifestyle in older patients [37] and healthy lifestyles among university students [38]. In the present study, this method was applied to recruit undergraduate students at Chengdu University of Information Technology (CUIT), Sichuan Province, between January and May 2024. All participants anonymously completed the survey using WenJuanXing (https://www.wjx.cn) (accessed on 10 January 2024), a widely used online questionnaire platform in China. The use of WenJuanXing leveraged students’ high level of digital engagement and has demonstrated improvements in data completeness and integrity in large-scale surveys [39]. Online research platforms represent a relatively new and efficient approach for collecting responses from large numbers of participants within a short period, at low cost, and from locations that are otherwise difficult to access [40]. More broadly, digital tools for research encompass a wide range of software and platforms designed to support different stages of the research process [41]. Recruitment was conducted through official university channels, including open elective courses, themed class sessions, counselor studio QQ groups, and campus outreach initiatives. The electronic informed consent form was embedded at the beginning of the questionnaire, and participants were required to read and agree to it before proceeding. In addition, forced-response settings ensured that all items were completed prior to submission, thereby eliminating item-level missing data.

### 2.2. Questionnaire Introduction

The survey instrument integrating both demographic items and the EPOCH questionnaire was used in this study [17]. Demographic variables were assessed using a structured questionnaire. Gender was coded as male or female. Grade level was categorized as freshman, sophomore, junior, or senior. Family structure was measured by whether the respondent was an only child or had siblings. Household registration distinguished between rural and urban backgrounds. Financial assistance was classified into four categories: (1) first class, targeting particularly vulnerable groups such as families with registered poverty status, families with disabled members, recipients of subsistence allowances, and orphans; (2) second class, referring to economically disadvantaged families not included in the first class; (3) third class, covering families with general financial difficulties; and (4) none, indicating no financial assistance, based on the Measures for the Administration of Student Financial Assistance Funds issued in 2019 and revised in 2021 [42,43]. Household income was divided into three levels: low income (annual household income below RMB 19,600, equivalent to USD 2706), middle income (RMB 19,600–27,000; USD 2706–3723), and high income (above RMB 27,000; USD 3727) [44].

This study employed the EPOCH Measure of Adolescent Well-being, available in both English [13] and Chinese [17]. The scale has demonstrated good internal consistency, with Cronbach’s α values ranging from 0.78 to 0.89. It has also shown strong reliability and validity in cross-cultural research, confirming its suitability for adolescent mental health studies across diverse cultural contexts [17,45,46]. Specifically, the EPOCH Scale consists of 20 items measuring five dimensions: Engagement, Perseverance, Optimism, Connectedness and Happiness. The questionnaire uses a Likert 5-point scoring system (1 = “Strongly Disagree” to 5 = “Strongly Agree”). Higher scores in each dimension indicate stronger manifestation of the corresponding psychological trait. Specifically, the scores for each dimension were calculated as the mean of the corresponding items (Engagement = mean of E1–E4, Perseverance = mean of P1–P4, Optimism = mean of O1–O4, Connectedness = mean of C1–C4, Happiness = mean of H1–H4).

### 2.3. Data Processing

Machine learning, a data analysis method that enables the automated detection of patterns in data [47], was applied using K-means cluster analysis to identify latent subgroups of university students with similar psychological well-being profiles. Importantly, demographic variables were not included in the clustering procedure and were examined in post hoc analyses to characterize the identified clusters. All analyses were implemented in Python 3.8.6 using the scikit-learn library [48]. To determine the optimal number of clusters, K-means clustering was performed for a range of values. For each value, the average Silhouette Coefficient was computed to evaluate the cohesion and separation of the resulting clusters [49]. This index quantifies the degree of similarity within clusters relative to other clusters, thereby guiding the selection of the most appropriate clustering solution.

## 3. Results

### 3.1. Overview of the Dataset

**Figure 1:**
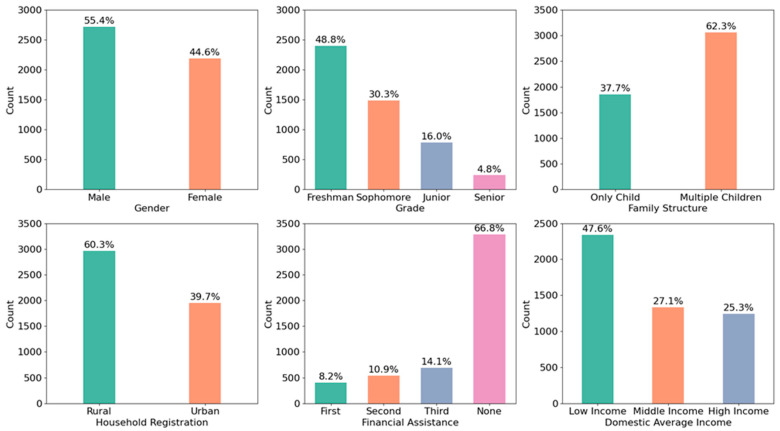
Distribution of Demographic Data (N = 4911).

A total of 4911 valid responses were collected from undergraduate students. Specifically, the study’s Cronbach’s alpha value was 0.952, which is high when compared to that of earlier research. Subscales also demonstrated satisfactory reliability: Engagement (α = 0.873), Perseverance (α = 0.822), Optimism (α = 0.838), Connectedness (α = 0.846), and Happiness (α = 0.917). The demographic distribution of the sample is presented in Figure 1. As shown, the gender ratio is relatively balanced. Freshmen represent the largest proportion of participants (48.80%), with a proportional decline in representation across higher academic years. Only 37.70% of respondents reported being only children, while students with rural household registration comprised over 60% of the sample. Additionally, the majority of participants (66.80%) indicated that they did not receive any form of financial assistance, and nearly half (47.60%) came from low-income households. Meanwhile, all demographic factors in the multiple regression analysis demonstrated statistically significant (*p* < 0.01) associations with psychological well-being [20].

The EPOCH questionnaire includes four items per dimension, presented in the same order as in the original version: Connectedness (C1, C2, C3, C4), Perseverance (P1, P2, P3, P4), Optimism (O1, O2, O3, O4), Happiness (H1, H2, H3, H4), and Engagement (E1, E2, E3, E4). The descriptive statistics for these items are summarized in Table 1.

In Table 1, we can observe that the Connectedness dimension showed a mean score of 3.63 ± 0.86, with item-level means ranging from 3.39 (C2) to 3.82 (C3). But the Perseverance dimension had a lower overall mean of 3.24 ± 0.81, with the lowest item mean observed for P4 (3.08 ± 1.01). The item-level scores for the three dimensions of Optimism, Happiness, and Engagement were relatively consistent, with average scores of 3.33 ± 0.89, 3.43 ± 0.90, and 3.31 ± 0.82, respectively, with H3 (3.55 ± 0.99) having the highest score.

Additionally, a correlation analysis was carried out to investigate relationships between demographic variables and psychological dimensions, as shown in Table 2.

In Table 2, correlation analysis revealed that demographic and socioeconomic factors were systematically associated with students’ psychological dimensions, although the effect sizes were generally small. Specifically, connectivity (r = 0.23, *p* < 0.001), optimism (r = 0.08, *p* < 0.001), and happiness (r = 0.12, *p* < 0.001) were all positively connected with gender, indicating that female students tended to report slightly higher levels in these areas. Grade level was negatively correlated with all dimensions, particularly happiness (r = −0.12, *p* < 0.001) and optimism (r = −0.10, *p* < 0.001), suggesting a decline in positive psychological traits as students’ grades increased through school, which is comparable to the findings reported by Xiao [50]. Only children showed higher scores across all five dimensions than students from multi-child families (r range: −0.05 to −0.07, all *p* < 0.001). Similarly, students with urban household registration demonstrated higher psychological scores compared with rural students (r range: 0.06–0.11, all *p* < 0.001). Finally, socioeconomic indicators, including financial assistance and family income, were positively associated with psychological well-being, with household income showing the strongest effects, particularly for connectedness (r = 0.18, *p* < 0.001) and happiness (r = 0.13, *p* < 0.001), which corresponds to the findings of Srivastava et al. [51].

### 3.2. K-Means Clustering Results

**Figure 2:**
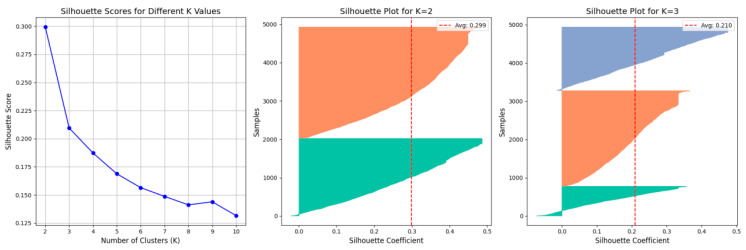
Cluster Evaluation for Different *K* Values.

To evaluate clustering performance and select the optimal number of clusters (*K*), the Silhouette Coefficient was applied [52], which is able to quantify cluster compactness and separation. We tested *K* values from 2 to 10 and calculated corresponding coefficients. The trend of Silhouette Coefficients across *K* values is shown in Figure 2 (left). Silhouette plots for *K* = 2 and *K* = 3 are displayed in Figure 2 (middle and right) to visualize cluster separation. The silhouette plot represents the partitioning of the data by assigning a unique color to each cluster, allowing for a visual assessment of its cohesion and separation.

As shown in the first subplot of Figure 2, the highest Silhouette Coefficient (0.3) occurs at *K* = 2, with values declining as *K* increases. Although a silhouette score of 0.3 is typically considered only moderate, the corresponding silhouette plot for *K* = 2 (Middle) reveals a visually clear and robust clustering structure that is superior to that of *K* = 3. Previous research has suggested that when the geometry of the cluster is non-spherical or the density is uneven, the evaluation effectiveness of silhouette score decline [53,54]. Specifically, in the second subplot of Figure 2, we observe that the silhouette coefficients for nearly all sample points are greater than zero, indicating that almost no instances were erroneously assigned to a cluster farther away than their own. Furthermore, the silhouette for each cluster forms a thick, relatively flat “inverted U” shape, suggesting that the assignment is consistent and stable for the vast majority of points within each cluster, rather than being driven by a few core samples. Most importantly, the two distributions are separated by a distinct gap near a silhouette coefficient of approximately 0.1, demonstrating clear separation between the two clusters.

Based on this analysis, the optimal *K* was 2, and students are divided two clusters. Cluster 0 includes 2018 samples (41.09%), and Cluster 1 includes 2893 samples (58.91%). Table 3 compares the average scores of both clusters across dimensions using independent samples *t*-tests to examine group differences. Statistical significance was assessed at *p* < 0.05, and effect sizes were calculated using Cohen’s d to quantify the magnitude of differences between clusters.

Table 3 demonstrates that Cluster 0 consistently and significantly outperformed Cluster 1 across all five dimensions of the EPOCH model, reflecting more positive psychological characteristics; thus, it can be categorized as the “high well-being group”. In contrast, Cluster 1 exhibited generally lower scores and is therefore identified as the “low well-being group”. Notably, all individual questionnaire items within each dimension showed statistically significant differences (all *p* < 0.001) with large effect sizes ranging from *d* = 1.167 to *d* = 1.887, indicating robust and consistent differentiation across all measured aspects of psychological wellbeing.

The most pronounced differences between the two clusters were observed in the dimensions of Happiness and Optimism, with exceptionally large effect sizes (Cohen’s *d* = 2.299 and *d* = 2.162, respectively) and score gaps of 1.37 and 1.31, suggesting these dimensions may serve as critical indicators for differentiating students’ psychological profiles. The next largest difference was found in Connectedness (*d* = 1.934), indicating notable disparities in interpersonal relationships. Although the differences in Engagement (*d* = 1.797) and Perseverance (*d* = 1.688) were comparatively smaller in terms of effect magnitude, both still represented large effects approaching or exceeding the conventional threshold for large effect sizes (*d* > 0.8), reflecting a considerable advantage for the high well-being group.

These findings suggest that the clustering model effectively distinguishes student subgroups with distinct psychological profiles and highlights the potential value of targeted mental health interventions, particularly focusing on enhancing happiness (where the largest effect was observed) and optimism among individuals in the low well-being group.

**Figure 3:**
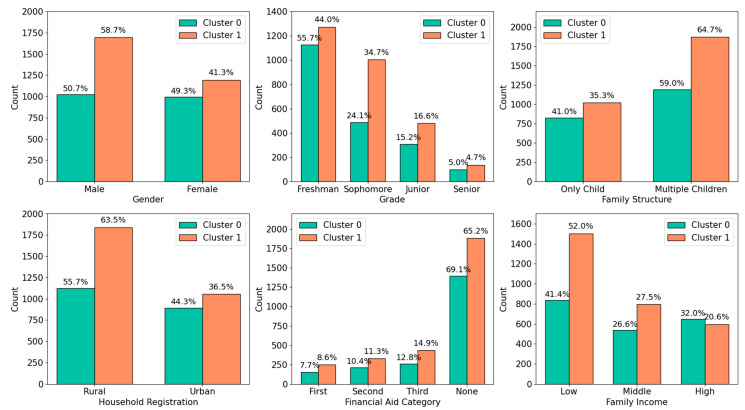
Demographic Differences Between Student Groups.

Further, to analyze demographic differences between the two clusters, we visualize the distribution of demographic factors under the cluster labels (Cluster 0 and Cluster 1), as shown in Figure 3.

As shown in Figure 3, significant differences exist in the distribution of demographic features between the clusters.

(1) Gender: Female students are evenly distributed between the two clusters, while Cluster 1 (low well-being group) dominates among males (62.3% male vs. 37.7% female in Cluster 1, compared to 48.5% male and 51.5% female in the overall sample). The probability of males being classified into the low well-being group is 36.5% higher than that of females.

(2) Grade: In Cluster 1(low well-being group), sophomores accounted for 45.1%, significantly exceeding the overall sophomore proportion (23.6%). The proportion of sophomores classified into Cluster 1 was 68.3%, compared to an average of 41.2% for other grades. This highlights sophomores’ uncertainty about their future direction and purpose, exacerbated by academic pressures and the need to choose a major [55].

(3) Family Structure: In Cluster 1 (low well-being group), multiple children account for 72.4% of the subgroup, compared to 27.6% of only children. This significant disparity in family structure suggests that parental resource allocation and support dynamics may influence cluster categorization. Only children receive high levels of parental attention and concentrated family resources, along with more patience and support, which enhances their prosocial behavior, thereby enhancing their sense of well-being [56].

(4) Household Registration: Rural students are significantly overrepresented in Cluster 1 (low well-being group) (78.3% rural vs. 60.2% rural in the overall sample), whereas urban students show a balanced distribution between clusters. In China, rural students face unique challenges, including heavy family economic burdens and limited parental support due to low education levels and insufficient emotional care [57].

(5) Financial Aid Category: Among students not receiving financial aid, 57.5% were classified into the negative psychological group (Cluster 1). In comparison, among those who received some form of financial assistance, the proportion assigned to Cluster 1 was even higher, exceeding 60%. This finding suggests that financial aid alone may not be sufficient to alleviate students’ psychological stress or emotional distress. Notably, within the subgroup of students who received the third class of financial aid, 62.6% were categorized into Cluster 1. This pattern may reflect these students are recognized but not fully addressed. These findings highlight the complexity of the relationship between financial aid and mental well-being, indicating that monetary support alone is insufficient.

(6) Family Income: Cluster 1 (low well-being group) is predominantly composed of low-income households (68.7% low-income in Cluster 1 vs. 47.6% in the overall sample). The probability of low-income students being classified into Cluster 1 is 61.2%, compared to 38.8% for middle- and high-income families. This aligns with financial aid patterns and underscores the socioeconomic challenges faced by this group.

In summary, individuals in Cluster 0 (positive-trait group) exhibit stronger social support, perseverance, optimism, happiness, and engagement, reflecting a healthier overall psychological state. Conversely, Cluster 1 (low well-being group) faces greater challenges in social connections, persistence, optimism, and life satisfaction. These findings highlight Cluster 1 as a vulnerable population requiring targeted mental health interventions and socioeconomic support to improve their well-being.

To further explore the psychological well-being challenges faced by 92 students of concern with overlapping factors (male, sophomore, multiple children, rural, received financial aid, low-income), we compared their EPOCH dimension scores against the overall sample. As summarized in Table 4, the students of concern demonstrated consistently lower scores across the five dimensions.

The comparative analysis revealed that the students of concern scored consistently lower across all five measured psychosocial dimensions compared to the overall sample, with all differences being statistically significant (*p* < 0.05). Specifically, the largest disparities were observed in happiness (Δ = −0.57, *p* < 0.001) and connectedness (Δ = −0.43, *p* < 0.001), followed by optimism (*p* < 0.001), perseverance (*p* = 0.002), and engagement (*p* = 0.016). These findings suggest that the students of concern may experience lower levels of emotional well-being, social support, and psychological resilience.

Meanwhile, we explore the psychological well-being of 108 well-being students with overlapping factors (female, freshman, only child, urban, high-income), and we compared their EPOCH dimension scores against the overall sample, as shown in Table 5.

The well-being students demonstrated significantly higher scores across all EPOCH dimensions compared to the general student population (all *p* < 0.05), with the largest disparities in Connectedness (Δ = 0.69, *p* < 0.001) and Happiness (Δ = 0.65, *p* < 0.001). Optimism (Δ = 0.44, *p* < 0.001) and Perseverance (Δ = 0.29, *p* < 0.001) also showed notable advantages, while Engagement had the smallest gap (Δ = 0.26, *p* = 0.005). These results suggest that socioeconomic and demographic factors may disproportionately enhance social connectedness and subjective well-being, highlighting potential intervention targets for broader student mental health promotion.

## 4. Discussion

The results showed that several demographic characteristics were significantly associated with lower levels of well-being. These included being male, a sophomore, from a rural area, from a multi-child family, from a low-income household, and dependent on financial aid. The most affected domains were Connectedness, Happiness, and Optimism. This pattern of psychological well-being may be overlooked by conventional aggregate-level methods. A two-cluster solution was validated by the Silhouette Coefficient (*K* = 2; score = 0.3), with silhouette plots indicating clear separation between groups. Cluster 0 (41.09%) exhibited consistently higher scores across all five EPOCH dimensions, while Cluster 1 (58.91%) demonstrated lower well-being levels, with the most pronounced disparities observed in Optimism (Δ = 1.31) and Happiness (Δ = 1.37). These findings provide direct support for our initial hypothesis. Distinct latent clusters of psychological well-being emerged among Chinese university students. They were differentiated by both EPOCH dimensions and sociodemographic characteristics.

Cluster 1 was characterized as the “low well-being group.” These students displayed significant psychosocial challenges, including weaker social support, diminished perseverance, a pessimistic outlook, reduced life satisfaction, and attentional disengagement. This two-cluster categorization facilitates more precise identification of students who may be at risk and provides a basis for developing subgroup-specific psychological support strategies. These findings echo prior research suggesting that reduced well-being and compromised mental health are associated with poorer academic performance and diminished achievement motivation in university students [58,59]. Students in Cluster 0 may benefit from a convergence of protective factors such as concentrated parental investment, urban educational resources, and better social capital.

These elements likely contribute to elevated levels of optimism and happiness, fostering greater resilience and engagement. Such profiles reflect what Seligman in 2011 termed “flourishing,” emphasizing not only the absence of distress but also the active presence of psychological strengths [60]. Recent studies show that flourishing has become an interdisciplinary focus across psychology, medicine, nursing, and related fields. Current research emphasizes happiness, health, virtue, and life satisfaction [61,62].

To further unpack these psychological patterns, it is important to consider how dispositional traits such as optimism influence students’ coping responses and goal-directed behaviors. Prior studies, such as the 2010 work of Carver et al., have shown that optimists are more likely to adopt adaptive coping strategies and remain persistent in the face of challenges [63]. These findings may partly explain the elevated levels of Optimism and Happiness observed in Cluster 0. More recent evidence from Tarrats-Pons in 2025 further supports this view, indicating that optimism encourages coping and enables individuals to more effectively overcome adversity [64]. This aligns with the broader literature emphasizing the role of psychological well-being in supporting academic functioning and personal growth [65,66].

Beyond individual traits, demographic patterns embedded in the clustering solution further illuminate the structural disparities underlying student well-being. The clustering solution revealed distinct well-being patterns across psychological and demographic dimensions. Cluster 1 was disproportionately male (62.3% vs. 37.7% female), sophomore-dominated (45.1% vs. 23.6% overall), and rural-origin (78.3% vs. 60.2% overall). Multiple children (72.4%) and low-income students (68.7%) were overrepresented, suggesting familial and socioeconomic factors compound psychological risks. Notably, financial aid recipients were more likely to be in Cluster 1 (>60%), suggesting that monetary support alone may be insufficient to address existing well-being disparities.

These demographic disparities should also be interpreted within the broader societal and policy shifts, particularly those related to family structure and the distribution of resources during childhood in China. In China, the adjustment of the One-Child Policy and the subsequent relaxation of fertility restrictions have led to the re-emergence of multi-child families and increasingly diverse family structures [67]. These evolving family dynamics, shaped by decades of population control policies, have contributed to heterogeneous outcomes in adolescent mental health [68,69,70]. Levels of parental attention, focused family resources, and more patience and support are given to only children, which improves their prosocial conduct and sense of well-being [56,71]. According to research by Wolke and Skew in 2012 [72], only children may be happier because they are free from sibling rivalry and arguments, which reduces psychological stress. Evidence from Liu and Jiang in 2021 further supports this view, showing that only children often enjoy higher-quality parent–child relationships, stronger emotional bonds with their parents, and more frequent communication [73]. This dynamic may partly explain why multiple children in our study exhibited lower levels of well-being across all psychological dimensions.

Further analysis of subtypes within financial aid recipients revealed important nuances. Among students receiving the third-tier financial aid, 62.6% were classified into Cluster 1, indicating that while their financial difficulties were formally recognized, their broader psychosocial needs may not have been adequately addressed.

This finding builds upon our earlier study and adds depth through a cluster-based lens, highlighting the disconnect between recognition and actual support impact.

This finding indicates that financial support without complementary psychosocial resources remains inadequate for improving student well-being. Prior research has shown that financial stress is closely linked to adverse mental health outcomes, with many students reporting food and housing insecurity as well as psychological distress [74,75,76]. Financial difficulties have been shown to be critical factors influencing student engagement [77,78], and insufficient support in this regard may contribute to both academic disengagement and psychological strain. Recent evidence suggests that financial stress can hinder students from fully benefiting from higher education by undermining both academic and social engagement [79]. Similarly, students who lost a need- and merit-based scholarship due to performance requirements were more likely to drop out, whereas those who retained such aid were more likely to graduate on time [80]. These findings imply that the design of financial aid programs may inadvertently create additional pressure rather than relieve it. Moreover, studies have emphasized that students’ psychological needs for developmental support are often overlooked in the implementation of financial aid policies [81]. In the Chinese context, Qi noted that, although national grants target students with financial difficulties, the level of support is often insufficient to cover actual living expenses, thereby limiting their effectiveness in enhancing student well-being [82]. Taken together, this evidence underscores the importance of integrating financial aid with psychological and developmental support to improve student well-being.

Notably, further examination uncovered two contrasting subgroups that highlight the compounding effects of demographic and socioeconomic variables. The subgroup of concern within Cluster 1 (n = 92)—primarily male sophomores from rural, low-income, multi-child families reliant on financial aid—scored significantly lower than their peers, particularly in Connectedness (Δ = −0.57) and Happiness (Δ = −0.43). In contrast, the high well-being subgroup within Cluster 0 (n = 108), mainly consisting of female freshmen from urban, affluent, only-child families, showed significantly higher scores, especially in Connectedness (Δ = 0.69) and Happiness (Δ = 0.65).

Existing research consistently demonstrates a positive relationship between psychological well-being and academic outcomes. For instance, Cárdenas found that higher well-being predicted better academic scores after several months [78]. Recent studies also show that subjective well-being practices are important for sustaining students’ long-term schooling-related quality of life [83]. These associations suggest that diminished well-being, as observed in this study’s vulnerable subgroup, may elevate the risk of academic underperformance and dropout. Moreover, demographic patterns reinforce this concern. National data show that male students are more likely to leave university prematurely than female peers [84,85]. Research also indicates that students from lower socioeconomic backgrounds face widening achievement gaps, particularly in language-related subjects [86,87]. These gaps between low- and high-income students require urgent action to ensure equal educational opportunities [88]. These patterns mirror our findings, where male, low-income, and rural students were overrepresented in the low well-being cluster.

The contrasting scores in Connectedness and Happiness between the subgroup of concern within Cluster 1 and the high well-being subgroup within Cluster 0 underscore the dual importance of emotional resilience and interpersonal ties in shaping student well-being. Just as a healthy sense of belonging contributes to positive life outcomes [89], strong interpersonal relationships provide greater social support [90], Conversely, weaker interpersonal ties are associated with lower levels of happiness [91] Among intervention strategies, peer mentoring stands out as particularly effective in fostering social connectedness and emotional resilience [92]. Recent evidence also shows that peer relationships are significantly associated with subjective well-being trajectories [83] and that peer support programs provide informational and psychosocial support that help reduce social isolation [93]. Such peer-based approaches have further been shown to enhance compassion, emotional literacy, and overall well-being [94,95].

Students from rural, low-income, and multiple-child backgrounds were overrepresented in the low well-being cluster, reflecting structural disadvantages in education [81,82]. Beyond socioeconomic factors, gendered norms further shape students’ psychological coping. Studies show that young men, constrained by societal expectations of emotional stoicism, are less likely to seek psychological support even when distressed [96]. By contrast, women are more likely to seek emotional support [97,98], with recent evidence indicating that men predominantly rely on problem-focused coping, whereas women tend to adopt emotion-focused strategies such as seeking support and expressing feelings [99,100].

Limited access to healthcare remains a critical issue in rural communities. In New Zealand, rural residents often have fewer locally available health services [101]. In China, research highlights the unequal distribution of healthcare resources between urban and rural areas [102,103]. Rural students also face restricted access to quality education, which undermines their academic performance and contributes to psychological difficulties [104]. These infrastructural limitations interact with cultural norms that emphasize emotional stoicism and discourage expressions of vulnerability. Consequently, male and rural students often encounter compounded cultural and structural barriers that reduce both their willingness and their ability to seek psychological support. These intersecting challenges, which involve internalized stigma, limited access to mental health services, and rigid social expectations, may help explain their consistently lower scores on well-being dimensions such as Connectedness and Happiness observed in this study. In addition to connectedness and happiness, optimism emerged as the most pronounced differentiator between the clusters (Δ = 1.31). This finding aligns with growing evidence that dispositional optimism plays a central role in emotional resilience and mental health. Recent studies indicate that fostering optimism in university students can effectively reduce depressive symptoms, particularly in contexts of socioemotional vulnerability [105]. Empirical work further demonstrates that optimism alleviates depressive symptoms [106]. Taken together, these findings suggest that the elevated levels of optimism observed in Cluster 0 may function as both a psychological resource and a protective factor, whereas its relative absence in Cluster 1 may contribute to greater emotional vulnerability and academic disengagement.

## 5. Conclusions

In line with our stated hypothesis, this study confirmed the existence of distinct latent clusters of psychological well-being among Chinese university students, differentiated by both EPOCH dimensions and sociodemographic characteristics. Using K-means clustering, we identified two clear profiles, with demographic and socioeconomic vulnerabilities linked to lower levels of Connectedness, Happiness, and Optimism. Male, rural, low-income students from multi-child families were disproportionately represented in the low well-being group, whereas urban, affluent, only-child female students consistently reported higher well-being, likely benefiting from protective resources such as concentrated parental support and enriched educational opportunities.

These contrasting profiles emphasize the need for targeted, evidence-based interventions, including peer mentoring, resilience training, and emotional literacy programs, alongside the integration of psychosocial resources with financial assistance, to promote flourishing and reduce inequalities in student well-being.

## 6. Limitations

Several methodological limitations should be acknowledged. First, the use of convenience sampling from a single university restricts the representativeness of the sample, limiting the generalizability of the findings to broader populations. Second, the cross-sectional design precludes causal inference, as the observed associations between sociodemographic characteristics and well-being profiles may reflect correlational rather than directional effects.

## 7. Future Research Directions

Given that this study relied on convenience sampling from a single university, future research should employ multi-site or cross-provincial sampling to improve representativeness and generalizability. Longitudinal designs would further help capture developmental changes over time and clarify potential causal mechanisms linking sociodemographic vulnerabilities with well-being trajectories.

## Figures and Tables

**Table 1:** Dimension Score Comparison for Items.

Dimensions	Questions				Total (Mean ± SD)
Connectedness	C1	C2	C3	C4	3.63 ± 0.86
	3.52 ± 1.12	3.39 ± 1.0	3.82 ± 0.99	3.77 ± 1.04	
Perseverance	P1	P2	P3	P4	3.24 ± 0.81
	3.42 ± 0.95	3.16 ± 1.04	3.31 ± 0.96	3.08 ± 1.01	
Optimism	O1	O2	O3	O4	3.33 ± 0.89
	3.27 ± 1.06	3.28 ± 1.13	3.39 ± 1.1	3.37 ± 1.01	
Happiness	H1	H2	H3	H4	3.48 ± 1.03
	3.38 ± 1.0	3.31 ± 1.01	3.55 ± 0.99	3.48 ± 1.03	
Engagement	E1	E2	E3	E4	3.31 ± 0.82
	3.39 ± 0.98	3.31 ± 0.96	3.32 ± 0.95	3.22 ± 0.97	
Note: Standard deviation = SD					

**Table 2:** Correlation Analysis of Demographic Variable and Psychological Dimensions.

Variable	Connectedness	Perseverance	Optimism	Happiness	Engagement
Gender					
Correlation value	0.23	0.00	0.08	0.12	0.01
*p*-value	<0.001	0.941	<0.001	<0.001	0.657
Grade					
Correlation value	−0.07	−0.04	−0.10	−0.12	−0.08
*p*-value	<0.001	<0.001	<0.001	<0.001	<0.001
Only Child					
Correlation value	−0.07	−0.05	−0.06	−0.05	−0.05
*p*-value	<0.001	<0.001	<0.001	<0.001	<0.001
Household registration					
Correlation value	0.11	0.06	0.09	0.07	0.06
*p*-value	<0.001	<0.001	<0.001	<0.001	<0.001
Financial					
Correlation value	0.07	0.02	0.05	0.06	0.04
*p*-value	<0.001	0.161	0.004	<0.001	0.15
Domestic average income					
Correlation value	0.18	0.11	0.12	0.13	0.11
*p*-value	<0.001	<0.001	<0.001	<0.001	<0.001

**Table 3:** Dimension Comparison Between Two Clusters.

Dimensions	Questions				Total
Connectedness	C1	C2	C3	C4	Mean ± SD
Cluster 0	4.21 ± 0.80	4.13 ± 0.62	4.53 ± 0.40	4.50 ± 0.46	4.34 ± 0.30
Cluster 1	3.04 ± 1.03	2.88 ± 0.62	3.33 ± 0.81	3.26 ± 0.88	3.13 ± 0.46
*p*-value	<0.001	<0.001	<0.001	<0.001	<0.001
Effect Size	*d* = 1.201	*d* = 1.586	*d* = 1.495	*d* = 1.467	*d* = 1.934
Perseverance	P1	P2	P3	P4	Mean ± SD
Cluster 0	4.00 ± 0.67	3.77 ± 0.99	3.95 ± 0.76	3.71 ± 0.91	3.86 ± 0.47
Cluster 1	3.01 ± 0.67	2.73 ± 0.68	2.87 ± 0.55	2.64 ± 0.62	2.81 ± 0.32
*p*-value	<0.001	<0.001	<0.001	<0.001	<0.001
Effect Size	*d* = 1.205	*d* = 1.167	*d* = 1.359	*d* = 1.245	*d* = 1.688
Optimism	O1	O2	O3	O4	Mean ± SD
Cluster 0	4.08 ± 0.70	3.97 ± 0.98	4.22 ± 0.74	4.14 ± 0.60	4.10 ± 0.38
Cluster 1	2.70 ± 0.64	2.80 ± 0.92	2.82 ± 0.73	2.83 ± 0.60	2.79 ± 0.36
*p*-value	<0.001	<0.001	<0.001	<0.001	<0.001
Effect Size	*d* = 1.703	*d* = 1.199	*d* = 1.641	*d* = 1.701	*d* = 2.162
Happiness	H1	H2	H3	H4	Mean ± SD
Cluster 0	4.16 ± 0.54	4.12 ± 0.60	4.36 ± 0.44	4.31 ± 0.50	4.24 ± 0.33
Cluster 1	2.84 ± 0.58	2.75 ± 0.54	2.98 ± 0.60	2.90 ± 0.61	2.87 ± 0.37
*p*-value	<0.001	<0.001	<0.001	<0.001	<0.001
Effect Size	*d* = 1.764	*d* = 1.820	*d* = 1.887	*d* = 1.877	*d* = 2.299
Engagement	E1	E2	E3	E4	Mean ± SD
Cluster 0	4.03 ± 0.70	3.97 ± 0.67	3.98 ± 0.67	3.87 ± 0.81	3.96 ± 0.44
Cluster 1	2.94 ± 0.65	2.85 ± 0.59	2.86 ± 0.56	2.77 ± 0.53	2.86 ± 0.34
*p*-value	<0.001	<0.001	<0.001	<0.001	<0.001
Effect Size	*d* = 1.336	*d* = 1.420	*d* = 1.432	*d* = 1.362	*d* = 1.797

**Table 4:** Comparison of EPOCH Dimension Scores Between the Students of Concern and Overall Students.

Dimensions	Mean Score (Students of Concern)	Mean Score (Overall Students)	Mean Difference	*p*-Value
Engagement	3.10 ± 0.80	3.31 ± 0.82	−0.21	0.016
Perseverance	2.99 ± 0.78	3.24 ± 0.81	−0.25	0.002
Optimism	3.02 ± 0.86	3.33 ± 0.89	−0.31	<0.001
Connectedness	3.20 ± 0.81	3.63 ± 0.86	−0.43	<0.001
Happiness	3.21 ± 0.80	3.78 ± 0.92	−0.57	<0.001

**Table 5:** Comparison of Mental Health Dimension Scores Between the Well-Being Students and Overall Students.

Dimensions	Mean Score (Well-Being Students)	Mean Score (Overall Students)	Mean Difference	*p*-Value
Engagement	3.57 ± 0.93	3.31 ± 0.82	+0.26	0.005
Perseverance	3.53 ± 0.88	3.24 ± 0.81	+0.29	<0.001
Optimism	3.77 ± 0.9	3.33 ± 0.89	+0.44	<0.001
Connectedness	4.32 ± 0.73	3.63 ± 0.86	+0.69	<0.001
Happiness	4.43 ± 0.86	3.78 ± 0.92	+0.65	<0.001

## Data Availability

The datasets used and/or analyzed during the current study are available from the corresponding author on reasonable request.
